# Targeted Chemotherapy of Glioblastoma Spheroids with an Iontronic Pump

**DOI:** 10.1002/admt.202001302

**Published:** 2021-04-12

**Authors:** Linda Waldherr, Maria Seitanidou, Marie Jakešová, Verena Handl, Sophie Honeder, Marta Nowakowska, Tamara Tomin, Meysam Karami Rad, Tony Schmidt, Joachim Distl, Ruth Birner‐Gruenberger, Gord von Campe, Ute Schäfer, Magnus Berggren, Beate Rinner, Martin Asslaber, Nassim Ghaffari‐Tabrizi‐Wizsy, Silke Patz, Daniel T. Simon, Rainer Schindl

**Affiliations:** ^1^ Gottfried Schatz Research Center – Biophysics Medical University of Graz Graz 8010 Austria; ^2^ Laboratory of Organic Electronics Department of Science and Technology Linköping University Norrköping 60174 Sweden; ^3^ Department of Neurosurgery Medical University of Graz Graz 8010 Austria; ^4^ Diagnostic and Research Institute of Pathology Medical University of Graz Graz 8010 Austria; ^5^ Institute of Chemical Technologies and Analytics Technische Universität Wien Vienna 1060 Austria; ^6^ Division of Biomedical Research Medical University of Graz Graz 8036 Austria; ^7^ Otto Loewi Research Center – Immunology and Pathophysiology Medical University of Graz Graz 8010 Austria

**Keywords:** electrophoretic drug delivery, gemcitabine, glioblastoma multiforme, organic electronic ion pumps

## Abstract

Successful treatment of glioblastoma multiforme (GBM), the most lethal tumor of the brain, is presently hampered by (i) the limits of safe surgical resection and (ii) “shielding” of residual tumor cells from promising chemotherapeutic drugs such as Gemcitabine (Gem) by the blood brain barrier (BBB). Here, the vastly greater GBM cell‐killing potency of Gem compared to the gold standard temozolomide is confirmed, moreover, it shows neuronal cells to be at least 10^4^‐fold less sensitive to Gem than GBM cells. The study also demonstrates the potential of an electronically‐driven organic ion pump (“GemIP”) to achieve controlled, targeted Gem delivery to GBM cells. Thus, GemIP‐mediated Gem delivery is confirmed to be temporally and electrically controllable with pmol min^−1^ precision and electric addressing is linked to the efficient killing of GBM cell monolayers. Most strikingly, GemIP‐mediated GEM delivery leads to the overt disintegration of targeted GBM tumor spheroids. Electrically‐driven chemotherapy, here exemplified, has the potential to radically improve the efficacy of GBM adjuvant chemotherapy by enabling exquisitely‐targeted and controllable delivery of drugs irrespective of whether these can cross the BBB.

## Graphical abstract

An electronically‐driven ion pump is engineered for the targeted delivery of the charged chemotherapeutic gemcitabine to brain cancer cells. Delivery of drug ions over a cation exchange membrane at currents in the nanoampere range triggers brain cancer cell death and microtumor disintegration. Local gemcitabine treatment offers a therapeutical window, in contrast to standard treatment.

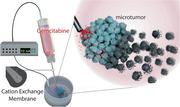

## 1 Introduction

Glioblastoma multiforme (GBM) is the most aggressive of all brain tumors, with a median survival of only 15 months after diagnosis.^[^ ^1^ ^]^ Treatment typically comprises maximal safe resection, followed by chemotherapy with BBB‐passing alkylating agents (e.g., temozolomide, TMZ) and radiotherapy (RT).^[^ ^2^ ^]^ Full resection is, however, rarely feasible^[^ ^3^ ^]^ due to infiltration of surrounding normal tissue. Residual tumor cells are, moreover, in 50% of cases, resistant to the standard chemotherapeutic drug used to treat GBM patients.^[^ ^4^ ^]^ Tumors thus reoccur locally in up to 90% of cases despite these interventions,^[^ ^5^ ^]^ and fewer than 5% of patients survive 5 years after diagnosis.^[^ ^6^ ^]^

Circumnavigation of the BBB through localized delivery to the resection site could (i) significantly advance the effectiveness of GBM adjuvant chemotherapy and (ii) allow the use of existing chemo drugs unable to efficiently penetrate the BBB. Clinical attempts to achieve local chemotherapy to date, namely using drug‐soaked wafers placed in the post‐surgical cavity^[^ ^7^ ^]^ and catheter‐based delivery systems^[^ ^8^ ^]^ have, however, been beset by problems. These include, in particular, limited controllability, and the occurrence of adverse effects such as wound healing abnormalities, seizures, intoxication, and cerebral edema.^[^ ^9^ ^]^

A powerful alternative which could avoid such issues is offered by the novel iontronic technology of organic electronic ion pumps (OEIPs) which provides local and precise electrically‐controlled delivery of ions as signal carriers. OEIP delivery, which comprises electrophoresis through a cation or anion‐exchange membrane (CEM or AEM, respectively), ensures that the electronic current precisely corresponds to the ionic (drug) delivery current and thus provides precise dosage control.^[^ ^10^ ^]^

To date, this technology has been successfully applied to the delivery of neurotransmitters in vitro and in vivo,^[^ ^11^ ^]^ plant hormones,^[^ ^12^, ^13^ ^]^ and modulators of inflammation in vitro,^[^ ^14^ ^]^ though not, to our knowledge, to the delivery of anti‐cancerous chemotherapeutics. OEIP‐based delivery has the potential to create high drug concentrations in a controllable manner, precisely at the site of tumor resection with high spatiotemporal resolution. OEIP‐based delivery, furthermore, only delivers the transported ions to the targeted area, without liquid flow or bulk quantities of associated solvents and would therefore not adversely affect brain pressure and the local biochemical environment.

**Figure 1:**
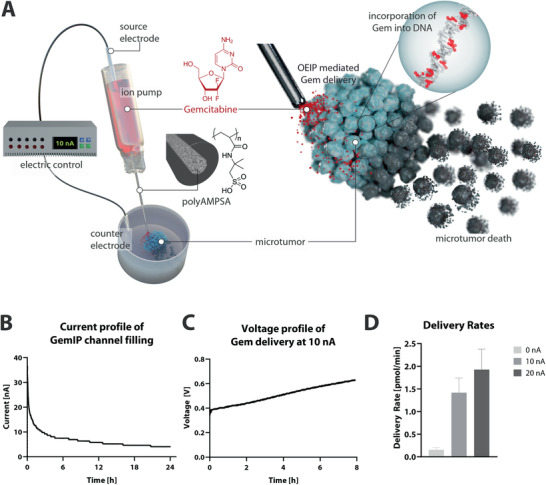
OEIPs as drug delivery tool for Gem to treat GBM microtumors in vitro. A) Illustrative image of an OEIP capillary channel in close vicinity of a GBM microtumor, which undergoes apoptosis and tumor destruction upon GemIP treatment, B) Time course of current recordings show channel loading over 24 h at a constant 0.5 V, C) Representative time course of voltage recordings of a GemIP device operated for 8 h at 10 nA, D) Delivery rates for Gem delivery via OEIPs at 0, 10, and 20 nA over 8 h (results shown as mean ± SEM, *n* = 5).

In the present study, we describe an in vitro evaluation of a miniaturized capillary fiber‐based OEIP for delivery of Gem, herein referred to as the gemcitabine ion pump (GemIP), designed to transport, or “pump”, the charged form of the chemotherapeutic Gem through a CEM “ion channel” toward target tumor cells (**Figure** **1**, details Figure S1a–d, Supporting Information). Gem is a cytostatic analogue of the nucleoside deoxycytidine,^[^ ^15^ ^]^ which causes S phase cell cycle arrest and consequently, programmed cell death following its incorporation into cellular DNA and disturbance of the cellular replication machinery (FDA, Ref. ID: 3 503 046).^[^ ^16^ ^]^ Gem is, moreover, a powerful radiosensitizer that potentiates treatment success in a combined therapy with radiation.^[^ ^17^ ^]^ Gem is widely used to treat aggressive cancers such as pancreatic, non‐small cell lung, and breast cancer via intravenous injection (FDA, Ref. ID: 3 503 046)^[^ ^15^, ^18^ ^]^ but is unsuitable for intravenous or oral treatment of GBM because it cannot cross the BBB to a sufficient extent.^[^ ^19^ ^]^

Our results first confirm the prediction that Gem is a chemotherapeutic with physical and chemical properties suitable for the administration via OEIP. Critically, OEIP‐mediated Gem delivery, moreover, killed GBM cell line monolayers and tumor cell spheroids, as efficiently as, or, in the latter case, more efficiently than “manual” Gem treatment, even to the extent of causing spheroid disintegration. Our study, thus, provides a solid basis and rationale for the development of novel iontronic implants for GBM treatment, utilizing this technology for the targeted delivery of a plethora of chemotherapeutics whose use is currently hampered by the BBB.

## 2 Electronically Controlled, Targeted Gem Delivery

We first aimed to prove that Gem can, indeed, be transported via capillary OEIPs. These devices are based on glass fiber capillaries, filled with a polyanion membrane material, 2‐acrylamido‐2‐methylpropane sulfonic acid, (AMPSA), as CEM, embedded in heat‐shrink tubes which serve as drug reservoirs (shown in Figure 1a). At acidic pH, Gem is predicted to be in its protonated state (strongest basic pKa 3.65), resulting in a positively charged form at low pH values (distribution of species shown in Figure S2A, Supporting Information).

Ion pump performance was initially tested with small cation K^+^ in the source at neutral pH and mounted on a target electrolyte. The two electrodes (source and counter) were arranged as in Figure 1a. The representative current–time course shows a stable current typically at 80–100 nA when 1 V was constantly applied to the system (Figure S1e, Supporting Information). Subsequently, the source solution was replaced with Gem at pH 4 (Figure 1a, red source solution). GemIPs were operated for 24 h at a constant voltage of 0.5 V to load the capillary channel with Gem (current trace in Figure 1b). The resulting decay of the current was due to the exchange of K^+^ in the channel with larger, sterically more demanding Gem^+^ cations. Indeed, the mobility of ions through the CEM is related to their diffusion coefficient, with higher diffusion coefficient corresponding to higher mobility, and thus higher conductivity.^[^ ^20^ ^]^ K^+^ has a diffusion coefficient of 1.957 × 10^−5^ cm^2^ s^−1^.^[^ ^21^ ^]^ Larger organic ions are generally closer to half that value, with some reports of cytidine monophosphate (chemically similar and only slightly larger than Gem) having a diffusion coefficient of around 0.62 × 10^−5^ cm^2^ s^−^.^[^ ^22^ ^]^ Due to the lower mobility of Gem, the resistivity of the channel increases and the current decreases with ongoing channel loading. It is noteworthy that the current approaches a current plateau after 6 h, but does not reach a constant current, possibly due to partial clogging of Gem in the CEM. Next, GemIPs were addressed with a constant current (10 and 20 nA) for 8 h (representative voltage profile of a GemIP operated at 10 nA for 8 h shown in Figure 1c). Here again the increase of channel resistivity shows in the increasing voltage over operation time, which is arguably due to steric hindrance of Gem in the CEM (see discussion section). The aromatic ring structure of Gem molecules allowed direct measurement of the concentration of OEIP‐delivered Gem via UV absorbance at 267 nm (Figure S2B,C, Supporting Information). Average delivery rates are 1.4 ± 0.3 to 1.9 ± 0.45 pmol min^−1^ for a constant current of 10 nA as well as 20 nA, respectively (Figure 1d). We also analyzed the rate of passive delivery (“leakage”) without electrical addressing. The mean passive Gem delivery rate was 0.15 ± 0.05 pmol min^−1^ and thus only 9 % of the active delivery flux (Figure 1d). Since a high concentration of H^+^ is present in the source electrolyte and the IEM is a cation exchange membrane, we also estimated the extent of potential H^+^ delivery. Electric operation for 24 h at a constant 10 nA current into a target of double distilled water (ddH_2_O), however, did not significantly change the pH of the GemIP target compared to the ddH_2_O control (Figure S2d, Supporting Information).

Electronically‐driven delivery of charged Gem via GemIPs is thus possible, and controllable by the applied voltage. As GemIP operation at 10 nA resulted in efficient delivery rates, we used this setting in all further GBM cell experiments.

## 3 Gem Kills GBM Cells More Efficiently Than the Treatment “Gold Standard” TMZ

**Figure 2:**
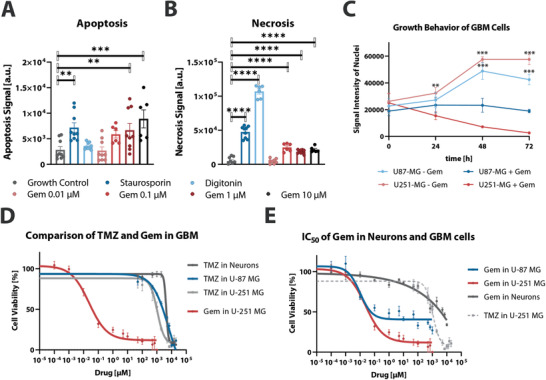
Gem‐induced effects in GBM cell lines U‐87 MG and U‐251 MG. A) Apoptosis signal in U‐87 MG induced by 1 µm staurosporine (dark blue), 50 µg mL^−1^ digitonin (light blue), 0.01 µm Gem (light red), 0.1 µm Gem (red), 1 µm Gem (dark red), and 10 µm Gem (black), compared with untreated growth control (grey). Results shown as mean ± SEM, *n* = 6–9 from two independent experiments, *p*‐value ≤ 0.01 (**), ≤ 0.001 (***), one‐way ANOVA. B) Necrosis signal in U‐87 MG induced by 1 µm staurosporine (dark blue), 50 µg mL^−1^ digitonin (light blue), 0.01 µm Gem (light red), 1 µm Gem (red), 10 µm Gem (dark red), compared with untreated growth control (grey). Results shown as mean ± SEM, *n* = 6–9 from two independent experiments, *p*‐value ≤ 0.0001 (****), one‐way ANOVA. C) Growth behavior of GBM cell lines U‐87 MG and U‐251 MG treated with or without 1 µm Gem. Results shown as mean ± SEM, *n* = 6 from two independent experiments, *p*‐value ≤ 0.01 (**), ≤ 0.001 (***), two‐sided *t*‐test. D) IC_50_ curves for treatment with TMZ for 72 h of U‐87 MG (blue), U‐251 MG (light grey), and primary neurons (dark grey). IC_50_ curve of Gem treatment for 72 h of U‐251 MG in red. For all data points, results shown as mean ± SEM, *n* = 6 from two independent experiments. E) IC_50_ curves for 72 h Gem treatment in U‐87 (blue), U‐251 MG (red), and primary neurons (dark grey). For easier comparison of the effective range of the Gem versus TMZ, the IC_50_ curve of U‐251 MG + TMZ is plotted in dashed grey lines. For all data points, results shown as mean ± SEM, *n* = 6 from two independent experiments.

The therapeutic potential of Gem in GBM was evaluated in vitro with the broadly‐used grade IV glioma cell line, U‐87 MG.^[^ ^23^ ^]^ Cells were manually treated with four different Gem concentrations (0.01, 0.1, 1, and 10 µm) and monitored for apoptosis and necrosis over 72 h (**Figure** **2**a,b). 1 and 10 µm Gem both induced strong apoptosis similar to the positive control, whilst cells treated with 0.01 and 0.1 µm Gem were unaffected (Figure 2a). The level of detected necrosis, in contrast, reached up to 23% of the respective positive control signal for all tested Gem concentrations (Figure 2b).

We subsequently (Figure 2c) monitored the effect of Gem treatment (1 µm) on GBM cell division by Hoechst dye nuclear staining using U‐87 MG cells and U‐251 MG GBM cell lines. U‐251 MG is another commonly used GBM cell line which differs from U‐87 MG in protein expression and migration and invasion behavior.^[^ ^24^ ^]^ While both untreated GBM cell lines exhibited two‐threefold increased growth within 72 h, Gem treatment significantly blocked cell division and even decreased GBM cell number (Figure 2c).

We next compared IC_50_ values of Gem and the “Gold Standard” GBM drug, TMZ, in order to directly compare their cell killing potentials (Figure 2d). Primary murine neurons and astrocytes, as well as U‐87 MG and U‐251 MG were treated with a broad range of concentrations of TMZ (Figure 2d, **Table** **1**) and Gem (Figure 2e) for 72 h. Strikingly, Gem IC_50_ was determined to be 10^5^‐fold lower than the TMZ IC_50_, for the tested GBM lines. Previous results also determined Gem IC_50_ values in a large set of glioblastoma cell‐lines in the sub‐µM range.^[^ ^25^ ^]^ TMZ IC_50_s for GBM and neurons were, moreover, within the same order of magnitude, indicating that the concentrations of TMZ required to kill GBM cells can also cause collateral damage to neurons. In marked contrast, nano and micromolar Gem concentrations effectively reduced the viability of GBM cells, whilst leaving neurons (Figure 2e, blue and red) and astrocytes (Figure S3, Supporting Information) unscathed. The latter were only negatively impacted by millimolar Gem concentrations (Figure 2e, grey). This apparent broad therapeutic window makes Gem an attractive candidate for locally‐applied chemotherapy in the brain.

**Table 1:** Comparison of IC_50_ values of Gem and TMZ in different GBM and healthy brain cell types after 72 h of drug treatment

Cell line	IC_50_	
	Gem [µm]	TMZ [mm]
U‐87 MG	0.01	6.4
U‐251 MG	0.02	1.4
Neurons	>1000	4.4
Astrocytes	68.7	–

## 4 Controlled Treatment with GemIPs Effectively Reduces GBM Cell Viability

**Figure 3:**
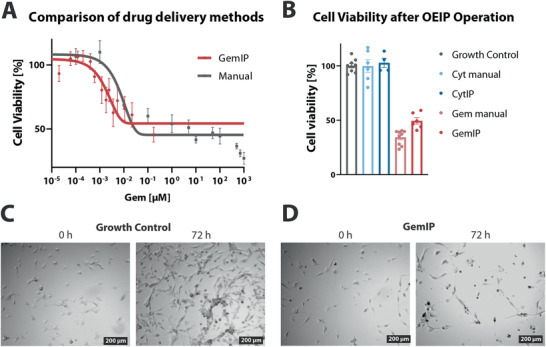
GemIP treatment of GBM monolayers. A) Comparison of dose‐dependence of manual and GemIP treatment in U‐87 MG cells; GemIPs were addressed at 10 nA const. for different time intervals; the concentration in the well was calculated based on mass spectrometric measurements. For manual treatment, *n* = 6 (two independent experiments), for GemIP treatment, n ≥ 10. Results shown as mean ± SEM. B) Cell viability of U‐87 MG treated with Cyt or Gem (50 µm manually, or OEIP operated at 10 nA for 8 h). Results shown as mean ± SEM, *n* = 4–8. C) Microscopic images of U‐87 MG cells growing for 72 h without treatment. D) Microscopic images of U‐87 MG cells, 72 h after GemIP treatment (operation for 8 h at 10 nA).

To be effective, GemIP based therapy should efficiently interfere with residual GBM tumor growth close to the resection site. To demonstrate the ability of GemIPs to reduce GBM cell viability, U‐87 MG cells were seeded in 96‐well plates and treated either manually (dosing control conditions), or with GemIP devices (**Figure** **3**a). GemIPs and PEDOT:PSS reference electrode were mounted in the cell‐containing wells without direct physical cell contact (setup in Figure 1a; and Figure S1c, Supporting Information) and operated at 10 nA for a specific amount of time, which enabled calculation of delivered Gem, based on Gem delivery amounts previously determined by mass spectrometry. Cell viability was then determined following a further 72 h incubation and plotted as a function of Gem concentration. Similar IC_50_ curves were obtained with GemIP delivery and manual drug administration (Figure 3a), indicating that GemIPs are a) able to induce GBM cell death via Gem delivery and b) precisely controllable, comparable to manual dosing.

To exclude toxic effects of OEIP operation per se or delivery of other toxic ions (e.g., H^+^), the above study was augmented to include IP‐mediated delivery of the non‐toxic analogue Cytidine (“CytIPs”, operated Figure 3b). Neither CytIP operation, nor manual Cyt treatment (50 µm) reduced cell viability (Figure 3b, blue). A significant reduction in cell viability to 49 ± 4% (Figure 3b, red), comparable to the effects of manual Gem dosing (37 ± 1% cell viability, Figure 3b, light red) was only observed with GemIP. Representative images of untreated and GemIP‐treated U‐87 MG cells at t_0_ and after 72 h (Figures 3c,d, respectively) clearly highlight the reduced viability associated with electronically‐controlled Gem delivery (Figure 3d).

## 5 GemIP‐Treatment Interferes with GBM Spheroid Cohesion and Viability

**Figure 4:**
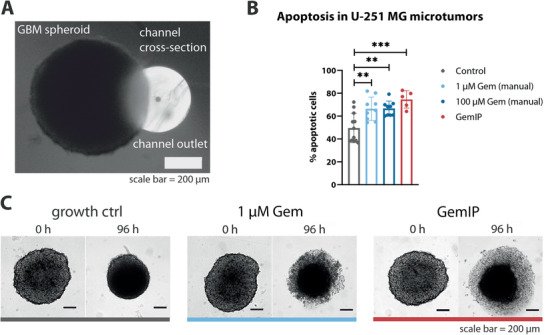
GemIP treatment of U‐251 MG in vitro microtumors. A) Size comparison of an U‐251 MG spheroid (black) next to an OEIP device (white, cross‐section), B) FACS analysis of apoptosis induced by manual treatment with Gem (1 or 10 µm) or with GemIPs operated for 24 h at 10 nA. Results shown as mean ± SEM, *p*‐value ≤0.01 (**), ≤0.001 (***), one‐way ANOVA. C) Visual comparison of spheroid cohesion at *t* = 0 h (after 30 h spheroid formation) and *t* = 96 h after treatment (from left to right: only media, 1 µm Gem applied manually and GemIP operated for 24 h at 10 nA).

In monolayer cell cultures, typically used to assess the potency of cytotoxic agents, all constituent cells grow in an elongated manner, and are consequently readily accessible to compounds dissolved in the cell medium. Native tumors, in contrast, contain many densely packed cells, in turn surrounded by a conglomerate of other cells. We reasoned that tumor cell spheroids derived from cultured GBM cells would, therefore, provide a more stringent test of GemIP efficacy (**Figure** **4**a, black spheroid next to a white cross‐section of an OEIP fiber capillary), the more so since they will also have other features of native tumors, such as a hypoxic core, cell–cell interactions and pH and nutrient supply gradients^[^ ^26^ ^]^ that render them physiologically predictive tools for clinical efficacy of anti‐cancer treatment procedures.^[^ ^27^ ^]^ Control FACS experiments revealed GBM microtumors to comprise 50 ± 12% average apoptotic cells (growth control). Positioning of GemIPs in the close vicinity of GBM microtumors (Figure 4a) and operation for 24 h at 10 nA, significantly increased the incidence of apoptosis above this baseline level judged by FACS analysis (75 ± 7% average apoptotic cells; Figure 4b). GemIP‐treatment, indeed, surpassed the efficacy of manual GEM treatment with 1 and 10 µm Gem (66 ± 10% and 67 ± 6% average apoptotic cells, respectively; Figure 4b). Microscopic observation of Gem‐treated spheroids revealed a disturbance of cell cohesion and spheroid decomposition, manifested as a halo of dead cells (Figure 4c, 1 µm Gem and GemIP). Control spheroids (Figure 4c, growth control), in contrast, became more compact over 96 h, without cell degression.

## 6 Conclusion

OEIPs allow precise, flow‐free delivery of bioactive substances such as neurotransmitters, with a very high degree of spatio‐temporal control.^[^ ^28^ ^]^ The present study evaluated the potential of this technology to electronically control the delivery of a chemotherapeutic drug, with a primary longer‐term application envisioned to be local adjuvant chemotherapy following GBM resection.

Gem, an efficacious chemotherapeutic that poorly crosses the BBB,^[^ ^19^ ^]^ was deemed a suitable candidate for electrophoretic transport through the ion pump as it is positively charged at low pH and small enough to be transported through the GemIP AMPSA CEM. Critically, GemIP‐mediated Gem delivery was confirmed to be temporally and electrically controllable with pmol min^−1^ precision. It, moreover, interfered with GBM cell viability as efficiently as manual Gem treatment, but with the major difference that the GemIP is electrically tuned. The observed toxicity could be fully ascribed to transported Gem and not, also, to parallel H^+^ transport or the ion pump set‐up itself, since no significant toxicity was associated with IP‐mediated transport of Cyt, Gem's non‐toxic “twin”.

GemIP‐mediated delivery also successfully induced GBM cell spheroid apoptosis, a key parameter of GBM treatment efficacy. Most strikingly, GemIP‐mediated delivery resulted in disintegration of the targeted spheroids. Since GBM tumor volume typically doubles within 50 days,^[^ ^29^ ^]^ eventually causing a fatal increase in brain pressure, timely efficient interference with this explosive growth is critically important.

Our study, found Gem to be a markedly more potent inhibitor of GBM cell line proliferation than TMZ, which is the standard drug for GBM chemotherapy. Importantly, our results also showed astrocytes and neurons to be, respectively, 10^4^ and 10^5^ orders of magnitude less sensitive to Gem, a reflection, we assume, of their post‐mitotic character. A corresponding therapeutic window was, in contrast, not observed with TMZ. These results are, moreover, consistent with studies in rats^[^ ^30^ ^]^ and non‐human primates,^[^ ^31^ ^]^ which showed convection‐enhanced delivery of Gem to the brain (max. 15 and 1.5 mm, respectively) to be well‐tolerated for up to 28 days. Taken together, these data support a therapeutic scenario in which a GemIP can be electrically adjusted to deliver local Gem doses that are harmless for neurons but which kill highly proliferative GBM cells. The potential for side‐effects following GemIP‐mediated local Gem delivery would be further reduced by exquisite drug targeting through outlets manufactured on a micrometer scale.

Local cancer therapy via electrical transport of Gem has been established for treatment of pancreatic cancer using iontophoretic devices,^[^ ^32^ ^]^ allowing us to compare these two different approaches. The iontophoretic device consists of an electrode in direct contact with a Gem drug reservoir surrounded by a polyurethane membrane as well as an inlet and outlet for drug flow‐through, combining electromigration, and electroosmotic forces.^[^ ^32^ ^]^ In comparison, our design utilizes a CEM for controlled Gem delivery and therefore avoids additional mechanical pressure or liquid flow for the necessary drug permeation. 2 mA currents applied to the iontophoretic device yielded a ninefold higher Gem concentration in ex vivo pancreatic tumors in comparison to passive diffusion and a marked shrinkage in an animal model.^[^ ^32^ ^]^ Instead, GemIPs are efficiently operated in a current range that is 100 000‐fold lower. This nanoamp range potentially prevents electrical stimulation of neuronal circuits.^[^ ^33^ ^]^ Instead, milliamp currents, when applied via deep brain stimulation, have been reported to cause adverse events such as myoclonic jerks, paresthesia, muscle contractions, and dyskinesias contralateral to the stimulated side.^[^ ^34^ ^]^

The present study thus shows that GemIP is a highly promising bioelectronic device that enables controlled local Gem administration and, thereby, the bypassing of the BBB, which currently severely constrains Gem treatment of GBM. Further developments will be needed to optimize this basic technology, for example, to improve pore size of the CEM and to overcome steric hindrance or π–π‐electron interactions between CEM and Gem molecules, that most likely underly increasing capillary channel resistance during extended operation. New ion exchange membranes or other fabrication technologies^[^ ^13^, ^35^ ^]^ may further reduce resistance build‐up and provide greater stability and versatility. Longer term, we envision a broader application of OEIP technology, for example, including the treatment of surgically inoperable tumors.

## 7 Experimental Section

##### OEIP Fabrication and Channel Filling

OEIPs were fabricated using glass fiber capillaries (25 µm internal diameter, 300 µm external diameter, Polymicro Technologies, CM Scientific, UK) as previously described in detail by Seitanidou et al.^[^ ^36^ ^]^ and stored in 1 mm KCl until use. They were then loaded by mounting them on Eppendorf tubes containing a reservoir of 100 µL deionized water. The OEIP source was 100 mm Gem adjusted to pH 4 with 0.1 mm HCl. Chemicalize, March 2019, https://chemicalize.com/, developed by ChemAxon, was used to predict Gem properties. For operation, in both the target and source electrolytes electrodes (PET sheet covered with poly(3,4‐ethylenedioxythiophene), polystyrene sulfonate (PEDOT:PSS)) were placed. Both electrodes were carbon‐coated to improve electrical contact. For channel loading, OEIPs were operated at a constant voltage (0.5 V) for 12 h.

##### Determination of Gem Delivery Rates and pH After Delivery

For measurement of the target Gem concentration and subsequent Gem delivery rate calculation, GemIPs were installed for operation into a target of ddH_2_O water and operated at constant currents (0, 10, 20 nA for 8 h). The Gem absorption spectrum was determined by measurement of UV absorption of 500 µm Gem between 200 and 400 nm with a CLARIOstar plate reader (BMG Labtech, Germany) and the Gem concentration in the target after GemIP operation was determined with a NanoDrop (Thermo Fisher Scientific, USA) at 267 nm. Delivery rate was calculated using the following equation:

(1) $$\begin{array}{c} delivery\;rate\left[mol/s\right]\;\;=\;\;\frac{Gem\;conc.\left[mol/L\right]*t\arg et\;\;volume\left[L\right]}{time\;\;of\;\;delivery\left[s\right]} \end{array}$$

For measurement of the target pH after GemIP operation, GemIPs were placed in a 100 µL ddH_2_O water target and operated for 24 h at 10 nA. Afterward, the GemIPs were dismounted and the pH of the aqueous target was measured with constant stirring using a HI 221 pH/ORP meter pH meter (Hanna Instruments, Germany) and an XS Sensor Micro S7 pH electrode (XS Instruments, Italy).

##### Determination of Gem Concentration by LC‐MS/MS

Chromatographic separation was performed on a Zorbax SB‐C18 column (50 mm x 4.6 mm, 1.8 µm, Agilent, USA) using a Dionex UltiMate 3000 system with a flow rate of 0.3 mL min^−1^ (solvent A: 0.1% formic acid in water; solvent B: 0.1% formic acid in acetonitrile). Injection volume was 2 µL and the applied gradient as follows: 0–10 min: 1% B–30% B; 10–13 min: 1% B. Chromatography was coupled to a TSQ mass spectrometer (Thermo Fisher Scientific, USA) operated with an electrospray ionization (ESI) source in positive mode. Selected reaction monitoring (SRM) mode was enabled with a parent mass of 264.100 Da for Gem and fragment masses of 94.980 and 112.000 Da. Peak integration and analysis was performed using the Thermo Xcalibur Quan Browser (version 3.0.63).

##### Cell Culture

U‐87 MG cells were cultivated in Eagle's minimum essential medium (E‐MEM) supplemented with 2 mm L‐glutamine, 1 mm sodium pyruvate, 0.1 mm non‐essential amino acids (NEAA), and 10% fetal bovine serum (FBS). U‐251 MG were cultivated in Dulbecco's modified Eagle's medium (D‐MEM) supplemented with 2 mm L‐glutamine and 10% FBS. All media and supplements were obtained from Thermo Fisher Scientific, USA. Primary neurons and astrocytes were freshly isolated from neonatal rat brain. Briefly, four cortical hemispheres were quickly removed from two PO‐P1 rat pups (sacrificed by decapitation), washed with phosphate buffer, crosswise‐chopped in 100 µm squares (McIlwain Tissue Chopper, Campden Instruments LTD, UK) and transferred to 1 mL Accutase for 20 min at 37 °C. Enzymatic digestion was stopped by addition of serum‐containing medium. The suspension was then filtered through a 0.4 µm cell strainer and centrifuged for 5 min at 300 g. For cultivation of neurons, cells were re‐suspended in cultivation medium (Neurobasal A Medium supplemented with 1% B‐27, 0.5 mm GlutaMAX (all Thermo Fisher Scientific, USA), 5 ng mL^−1^ β‐FGF and 20 ng mL^−1^ EGF (both PrePro Tech, USA), and containing 0.2% Normocin (Invivogen, USA). After 4 days, β‐FGF concentration was increased to 10 ng mL^−1^. Astroglial cultures were prepared using the same basic procedure with the following differences: Accutase digestion for 15 min at 37 °C and quenching by addition of L‐15 medium containing 10% FBS (Gibco, Thermo Fisher Scientific, USA), centrifugation for 5 min at 100 g, resuspension in D‐MEM containing 20% FBS, 2 mm GlutaMAX (all Gibco, Thermo Fisher Scientific, USA), 1 mm sodium pyruvate (Sigma–Aldrich, Germany), and 0.2% Normocin (Invivogen, USA), and cultivation in T‐25 flasks of the resultant mixed glial cultures until confluence (around day 10–11). Pure astrocytic cultures were then obtained by agitation in an incubator shaker at 200 RPM at 37 °C for 1 h to eliminate microglia and a further 24 h shaking to detach and so remove oligodendrocyte precursor cells.

##### Compound Preparation and Storage

Temozolomide was stored as a powder at 4 °C and weighed‐in and dissolved in the corresponding media immediately before use. Gemcitabine and Cytidine were stored as 100  mm aqueous stocks at −20 °C, Digitonin as 5 mg mL^−1^ aqueous stock at ‐20 °C, and Staurosporine as a 1 mm stock in DMSO at −20 °C. All compounds were obtained from Merck Millipore, USA.

##### Cell‐Based Assays

All assays were performed using flat‐bottomed, transparent (absorbance readouts) or black‐walled (fluorescence or luminescence readouts) 96‐well plates (Corning, USA) and a CLARIOstar plate reader (BMG Labtech, Germany).

For evaluation of apoptotic/necrotic cell death, U‐87 MG cells (4000 cells per well) were seeded 24 h prior to treatment with Gem (0.01, 0.1, 1, and 10 µm), staurosporin (1 µm, apoptosis positive control), and digitonin (50 µg mL^−1^, necrosis positive control). Apoptosis and necrosis were quantified using the RealTime‐Glo Annexin V Apoptosis and Necrosis Assay system (Promega, Germany), excitation wavelength 485 nm per emission wavelength 520–530 nm.

For cell proliferation studies, U‐87 MG cells and U‐251 MG cells (8000 cells perwell) were treated with 1 µm Gem or vehicle‐containing medium for 0, 24, 48, and 72 h followed by Hoechst staining (6 µg mL^−1^ Hoechst 33 258 in PBS, incubation for 10 min/RT/dark, followed by two PBS wash steps) and quantitation at excitation wavelength 350 nm permission wavelength 455 nm.

For Gem and TMZ dose‐inhibition studies, U‐87 MG cells (4000 cells perwell) and U‐251 cells (1500 cells per well) were treated for 72 h with a range of freshly prepared TMZ solutions (0, 50–20 000 µm) and Gem solutions (0, 0.0001–10 000 µm). Cell viability was determined by MTS assay (CellTiter 96 AQueous One Solution Cell Proliferation Assay Promega, Germany) according to the manufacturer's protocol. IC_50_ values were obtained by fitting data points to a sigmoidal curve using Prism 8 software (GraphPad, USA) and the following equation, in which top and bottom refers to the minimally and maximally inhibited response, respectively:

(2) $$\begin{array}{c} Y\;\;=\;\;Bottom+\;\;\frac{Top-Bottom}{1+{\left(\frac{IC_{50}}{x}\right)}^{HillSlope}} \end{array}$$

##### GemIP Treatment of GBM Cells

The effects of OEIP‐mediated Gem delivery on GBM cells were evaluated using U‐87 MG monolayers and U‐251 MG cell spheroids in 96 well plates. Monolayers were cultivated in flat‐bottom 96 well plates (Corning, USA) treated 24 h after seeding (4000 cells per well). Spheroids were generated by cultivating U‐251 MG cells (10 000 cells per well) in round bottom ultra‐low attachment spheroid microplates (Corning, USA) for 30 h. OEIPs (containing either Gem (100 mm Gem, pH 4, GemIP) or Cytidine (100 mm, pH 4, CytIP) in the source reservoir) and counter electrode (PEDOT:PSS with carbon coating) were mounted in individual wells with their tips immersed in the cell media without contacting the underlying cell monolayer and operated at 10 nA for different time periods (cell monolayers: 0, 10, 30, 60, 100, and 200 s; 5, 10, 15, 20, 25, 30, 45, 90, and 180 min; 24 h; spheroids: 24 h). Cell monolayers were afterward monitored both visually (4× magnification on an Olympus IX70 Diagnostic Instruments Inc. microscope, camera model 34.0, and Visitron Visiview Software) and by MTS assay (Promega, Germany) following incubation for a further 72 h cells. Treated spheroids were evaluated microscopically as described above 96 h after Gem treatment and for apoptotic cell death after 48 h using a FITC Annexin V Apoptosis Detection Kit I (BD Biosciences, USA) in accordance with the manufacturer's protocol. Measurements were performed on a CytoFLEX S (Beckmann Coulter, USA). Acquisition was terminated after 10 000 cell counts and follow‐up data analysis performed with CytoFLEX analysis software (Beckmann Coulter, USA).

##### Statistical Analysis

Statistical analysis was performed using commercially available software (Prism 8, GraphPad, USA), and specific statistical tests were used as stated in the text.

## Conflict of Interest

M.B. and D.T.S. are shareholders in the small, researcher‐controlled intellectual property company OBOE IPR AB (oboeipr.com), which owns the patents related to the ion pumps presented above. All other authors declare no conflict of interest.

## Author Contributions

L.W. and M.S. contributed equally to this work. R. S., L.W., M.S., M.J., S.P., and D.S. conceived of the ideas, supervised the project, interpreted the data, and wrote the manuscript. L.W. prepared the original draft of the paper and performed determination of delivery rates, observation of apoptosis, necrosis, cell growth, and IC_50_ in GBM cell lines, neurons, and astrocytes, GemIP treatment of U‐87 MG monolayers and observation of cell viability and GemIP treatment of U‐251 MG spheroids. M.S. and M.J. manufactured the OEIP devices. V.H. and S.P. performed FACS analysis of GBM spheroids. S.H. and T.T. performed MS measurements. M.N. prepared primary astrocytes. J.D. helped with determination of IC_50_ in GBM cell lines and observation of apoptosis and necrosis. T.S. took microscopic images and data analysis. R.B., M.B., B.R., and N.G. contributed to the study design, revised the manuscript, and supported data analysis together with all other authors.

## Supporting information

Supporting Information

## Data Availability

The data that support the findings of this study are available from the corresponding author upon reasonable request.
