# Supplementary material for: Targeted Chemotherapy of Glioblastoma Spheroids with an Iontronic Pump
Source: Adv Mater Technol. 2021 Apr 12;6(5):2001302. doi: 10.1002/admt.202001302 (PMC8218220; doi:10.1002/admt.202001302)
Supplement: Supplementary file 1 — Supporting Information [file ADMT-6-2001302-s001.pdf]

© 2021 Wiley-VCH GmbH

ADVANCED MATERIALS TECHNOLOGIES

Supporting Information

for *Adv. Mater. Technol.,* DOI: 10.1002/admt.202001302

Targeted Chemotherapy of Glioblastoma Spheroids with an Iontronic Pump

Linda Waldherr, Maria Seitanidou, Marie Jakešová, Verena Handl, Sophie Honeder, Marta Nowakowska, Tamara Tomin, Meysam Karami Rad, Tony Schmidt, Joachim Distl, Ruth Birner-Gruenberger, Gordvon Campe, Ute Schäfer, Magnus Berggren, Beate Rinner, Martin Asslaber, Nassim Ghaffari-Tabrizi-Wizsy, Silke Patz, Daniel T. Simon,* and Rainer Schindl**

Supporting Information

Targeted chemotherapy of glioblastoma spheroids with an iontronic pump

Linda Waldherr, Maria Seitanidou, Marie Jakešová, Verena Handl, Sophie Honeder, Marta Nowakowska, Tamara Tomin, Meysam Karami Rad, Tony Schmidt, Joachim Distl, Ruth Birner-Gruenberger, Gord von Campe, Ute Schäfer, Magnus Berggren, Beate Rinner, Martin Asslaber, Nassim Ghaffari-Tabrizi-Wizsy, Silke. Patz*, Daniel T Simon*, Rainer Schindl*

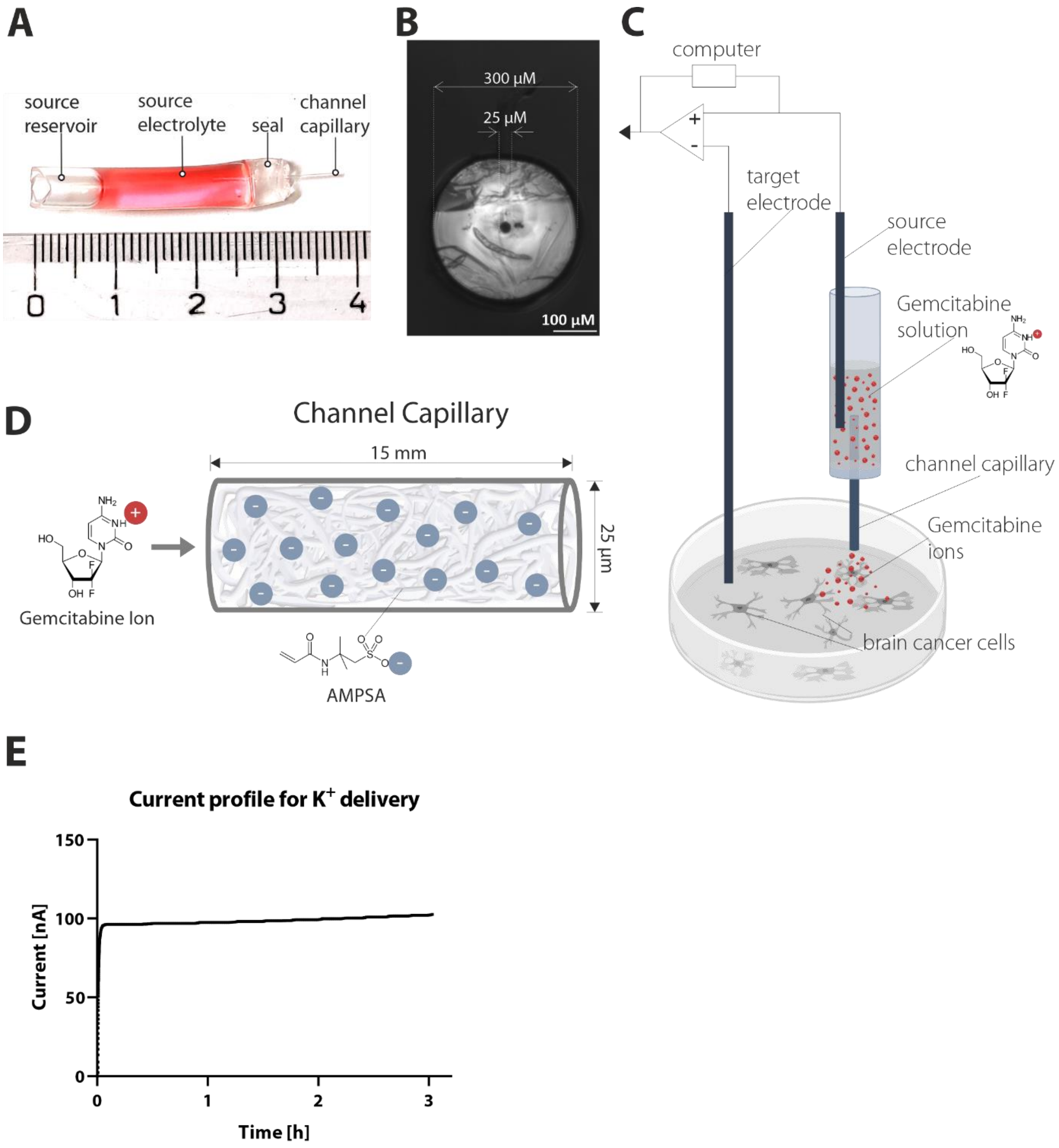

Figure S1. Capillary OEIPs in detail. (A) Photograph of an OEIP device, showing the source reservoir filled with the source electrolyte (red) and the seal spanned by the capillary channel, (B) Cross-section of the fiber capillary channel, showing the channel that is filled with the CEM with an internal diameter of 25 µm and an external diameter of 300 µm, (C) Schematic GemIP setup, as used in this work for the treatment of GBM cell monolayers and spheroids, showing the GemIP device dwelling in a cell culture dish, contacted with source and target electrode for electric controllability, (D) Schematic illustration of electromigration of a protonated Gem cation through the channel capillary, filled with AMPSA. (E) Representative time course of a current recording of an OEIP operated at const. 1 V for 3 h with a 10 mM KCl source.

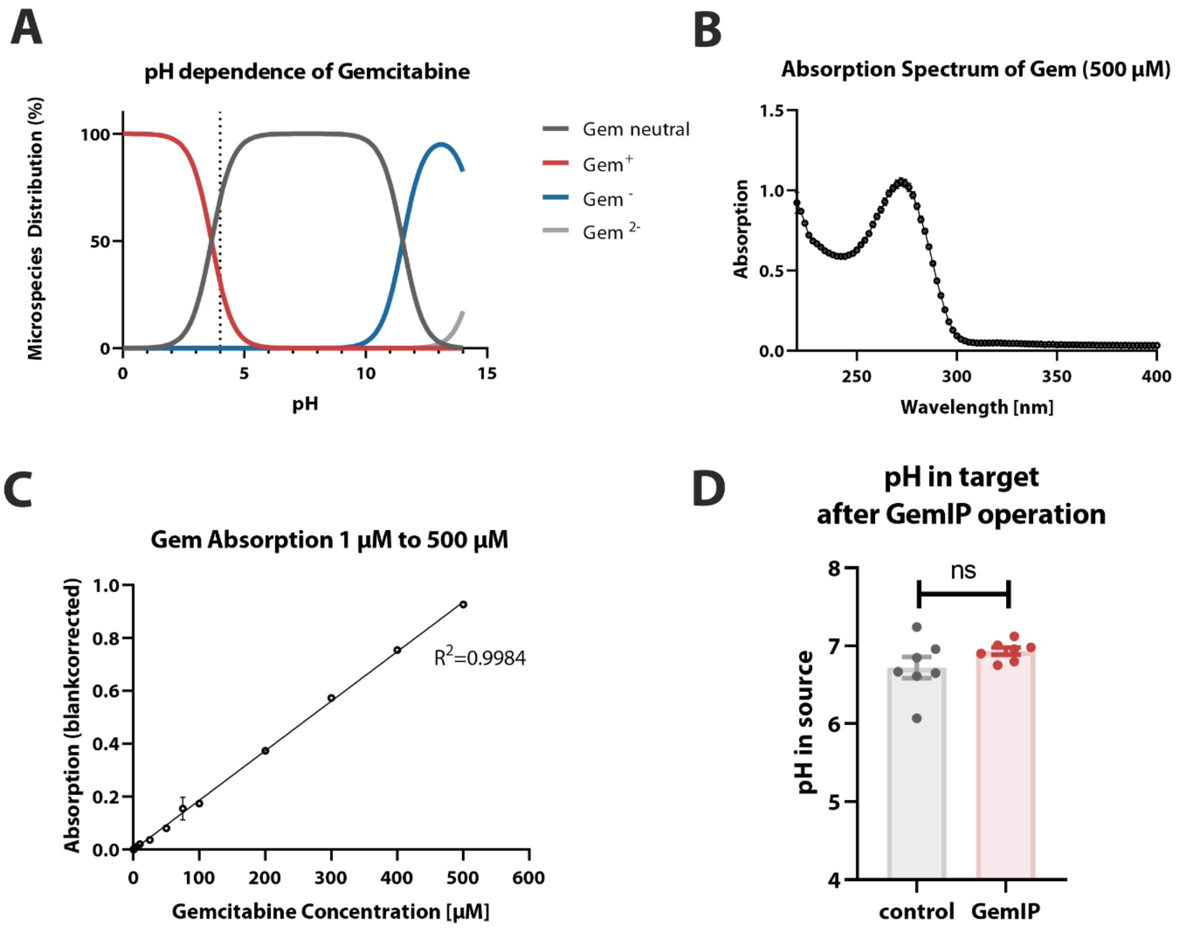

Figure S2. Detection of Gem concentration and pH in the target electrolyte. (A) pH-dependent distribution of differently charged Gem species, calculated via ChemAxon software chemicalize. In red, the species distribution of the single positively charged Gem is shown, **(B)** Absorption spectrum of aqueous Gem solution (500 µM), **(C)** Absorption linearity of Gem at 267 nm, **(D)** pH in target electrolyte (ddH_2O) after GemIP operation for 24 h at 10 nA (control = ddH_2O; n = 7, two-sided t-test, results shown as mean ± SEM).

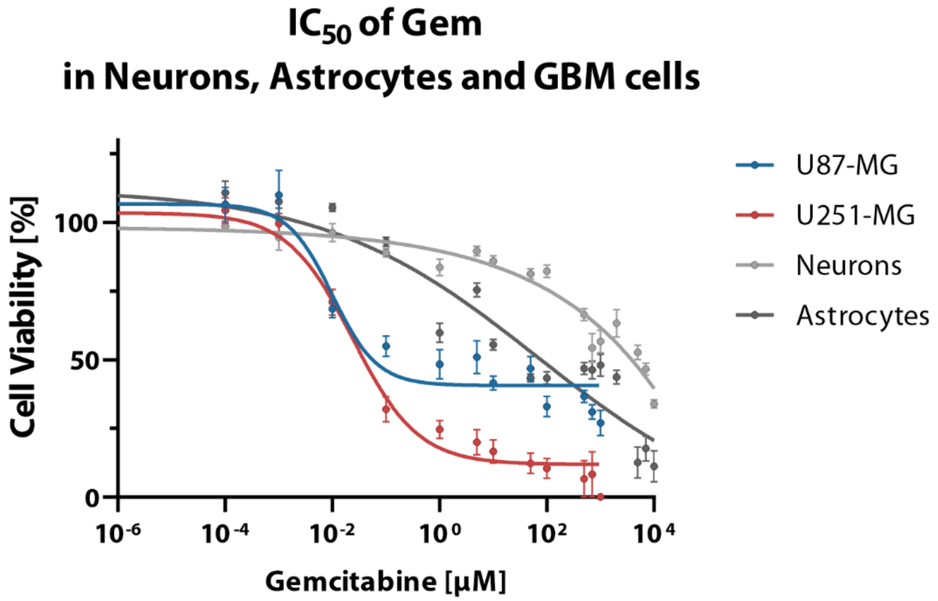

Figure S3. IC_{50} of Gem in Astrocytes in comparison to GBM cell lines and neurons. Cells were treated with Gem concentration range for 72 h (n ≥ 6 from 2 independent experiments, results shown as mean ± SEM).
